# Pediatric Traumatic Retinal Detachment: Clinical Features, Prognostic Factors, and Surgical Outcomes

**DOI:** 10.1155/2018/9186237

**Published:** 2018-06-25

**Authors:** Dilek Yaşa, Zeynep Gizem Erdem, Ufuk Ürdem, Gökhan Demir, Ali Demircan, Zeynep Alkın

**Affiliations:** Department of Ophthalmology, Beyoğlu Eye Research and Training Hospital, No. 2, Beyoglu, 34421 Istanbul, Turkey

## Abstract

**Purpose:**

We report the clinical characteristics, prognostic factors, and surgical outcomes for 23-gauge pars plana vitrectomy (23-G PPV) in pediatric cases of traumatic retinal detachment (RD).

**Methods:**

Medical records of pediatric patients who underwent 23-G PPV to treat traumatic retinal detachment were retrospectively reviewed. These patients underwent a follow-up examination at least 1 year following surgery. Associations between various preoperative factors and anatomical and visual outcomes were analyzed. An Ocular Trauma Score (OTS) and a Pediatric Ocular Trauma Score (POTS) were calculated for each patient. Raw scores were converted to their corresponding OTS and POTS categories. Final visual acuities by categories were compared with those in the OTS and POTS studies.

**Results:**

The mean age of the patients was 9 ± 4 years, and the male-to-female ratio was 4.7 : 1. The mean follow-up time was 23 ± 14 months. Anatomical success was achieved in 72% of the eyes, and functional success (>5/200) was achieved in 37% of the eyes. Functional success was less common among patients with visual acuities less than hand motion, macula-off retinal detachment, proliferative vitreoretinopathy at presentation, and recurrent retinal detachment during follow-up. When we compared the categorical distribution of final visual acuities in all categories, our results were significantly different than those suggested by OTS and POTS.

**Conclusions:**

Visual outcomes are poorer compared to anatomical outcomes. OTS and POTS do not provide reliable prognostic information if the patient has RD. Presenting visual acuity, the presence of macula-off RD, and PVR are all important predictors of final visual acuity.

## 1. Introduction

Retinal detachment is relatively uncommon in children, and the major cause of retinal detachment in pediatric populations is ocular trauma [1]. However, clinical features and surgical outcomes for traumatic retinal detachment patients in pediatric populations are not well defined in the literature. They are typically reported retrospectively as a part of all ocular injuries or all retinal detachment case studies, and, thus, are represented by a limited number of patients. Only Sarrazin et al. [2] and Sul et al. [3] evaluated pediatric traumatic retinal detachments as an independent group. As a result, there is a need for additional studies to better describe this subgroup.

Ocular trauma patients are not a homogeneous group, and they present with a great diversity of clinical features. As a result, it is difficult to predict functional outcomes after globe injuries. Kuhn et al. developed the Ocular Trauma Score (OTS) to predict visual outcomes at 6 months after injury [4]. However, the predictive value of the OTS after pediatric trauma is not clear. Acar et al. [5] proposed the Pediatric Ocular Trauma Score (POTS), but to the best of our knowledge, it has only been evaluated in two studies involving a limited number of patients [6, 7]. Studies evaluating the applicability of the OTS and POTS to pediatric cases include a limited number of cases with retinal detachment [5–8]. As a result, there is still a need for studies to evaluate the validity of the OTS and POTS in pediatric patients suffering from traumatic retinal detachment.

In this study, we report clinical features and surgical outcomes following 23-G PPV in a group of consecutive pediatric patients diagnosed with traumatic retinal detachment associated with open- or closed-globe trauma. In addition, we evaluate the prognostic value of several preoperative factors and the OTS and POTS in this patient group.

## 2. Methods

This study was conducted in accordance with the Declaration of Helsinki, and the approval was obtained from the institutional review board. Medical records of patients who underwent 23-G PPV in our clinic from 2013 to 2016 were retrospectively evaluated. Patients younger than 18 years old who underwent follow-up for at least 1 year were included in the study. Patient demographics, etiology, the type and zone of trauma, traumatic pathologies other than retinal detachment, the presence or absence of macular detachment, surgical procedures, preoperative and postoperative visual acuities, and complications were recorded.

Visual acuity tests were performed with an ETDRS chart or LEA symbols, depending on the age or cooperation of the patient. Visual acuities were divided into five groups similar to the OTS study: no light perception (NLP), light perception (LP), hand motion (HM), 1/200 to 19/200, 20/200 to 20/50, and 20/40 or better. Functional success was defined as the distance-corrected visual acuity of 5/200 or more. Anatomical success was defined as complete retinal attachment at the final visit.

The determination of the OTS and POTS is described in detail in the literature. First, raw OTS and POTS were calculated for each patient, and then, numerical values were converted to their corresponding OTS and POTS categories. If preoperative visual acuity was not measured, the OTS was not calculated. Finally, the similarity of final visual acuities by category was compared with those in the OTS and POTS studies.

### 2.1. Statistical Methods

Statistical analyses were performed using MedCalc for Windows, version 12.7.7 (MedCalc Software, Ostend, Belgium), and categorical variables were described by using frequencies (*n*) and percentages (%).

The chi-square (*χ*^2^) and chi-square exact (at *r* × *c* contingency tables) tests were used for categorical variables which were expressed as observation counts (and percentages). A two-tailed *p* value of less than 0.05 was considered statistically significant. Multivariate analysis included anatomical/functional success as the dependent variable. The parameters that were significantly associated with anatomical/functional success in bivariate analysis were included as independent variables in the multiple logistic regression analysis.

## 3. Results

The mean age of the patients was 9 ± 4 years, and the mean follow-up time was 23 ± 14 months. Demographics and trauma type are presented in Table 1. Most of the patients were male, and there was a male-to-female ratio of 4.7 : 1 (*p* < 0.001). Open-globe trauma was more common (*p*=0.008).

In patients with open-globe trauma, 17 (47%) had penetrating trauma, 12 (33%) had a globe rupture, and 7 (19%) had an intraocular foreign body (*p*=0.051, chi-square test, two-tailed). In this patient group, 29 (51%) patients had zone 1 trauma, 12 (21%) patients had zone 2 trauma, and 16 (28%) patients had zone 3 trauma (*p* < 0.016, chi-square test, two-tailed).

The most common cause of globe injury was an injury with a knife (Table 2). Injuries from a stone or a ball were also relatively common (Table 2). Associated ocular pathologies are presented in Table 3.

Anatomical success was achieved in 72% of the eyes by the final visit. Functional success (visual acuity ≥5/200) was achieved in 37% of the eyes. Preoperative and postoperative visual acuities are presented in Table 4. The association of several factors with postoperative anatomical and functional success is presented in Table 5. A multiple logistic regression analysis was performed for variables which were significantly associated with functional success (Table 5).

The POTS was calculated for all patients. However, preoperative visual acuities were not present in the medical records of five patients (aged 2 to 3 years old). The OTS was not determined for these patients. In addition, the presence or absence of a relative afferent pupillary defect (RAPD) was not recorded in the medical records of most patients. As a result, a RAPD was not considered when determining OTS. When we compared the categorical distribution of final visual acuities for all categories, our results were significantly different than those suggested by the OTS and POTS (Tables 6 and 7).

## 4. Discussion

In this study, we evaluated the clinical features and surgical outcomes after 23-G PPV in a group of consecutive pediatric patients suffering from traumatic retinal detachment associated with open- or closed-globe trauma. We divided patients into three age groups (0–6, 6–12, and 12–18 years). Although the 6–12-year age group had slightly more patients, the number of patients in each group was not statistically different. In agreement with the literature [2, 3], most of our patients were male (male-to-female ratio: 4.7 : 1), and open-globe trauma was more common (63% versus 37%). In pediatric cases involving retinal detachment, Sarazzin et al. [2] found that open-globe trauma was more common (62% versus 38%), and the male-to-female ratio was 5.2 : 1, which is very similar to our patient group.

The mechanism of traumatic open-globe injuries in adult and pediatric cases is usually well described [5, 9]. Acar et al. from Turkey reported in a series of pediatric open-globe traumas cases that the most common cause of injury was with a knife. Previous reports of pediatric traumatic retinal detachment have concentrated on clinical characteristics and surgical results. Thus, the mechanisms of injury in cases with traumatic retinal detachment are not well described. However, in this study, the mechanism of injury was recorded for all patients, and the most common cause of injury (30%) was with a knife. Also, it is noteworthy that although the mechanisms of injury were diverse, 59% of all injuries were caused either by a knife, a stone, or a ball (Table 2).

In this study, anatomical success was achieved in 72% of all eyes, and functional success (visual acuity ≥5/200) was achieved in 37% of all eyes by the final visit. Similar to previous reports in the literature, these outcomes are worse than those for retinal detachment in adults [2, 3, 5, 10]. The diversity of clinical features of trauma patients has led several investigators to study the factors that influence final visual acuity. In the multivariate model, we found that better visual acuity (>HM) at the time of presentation was associated with a better (>5/200) postoperative visual acuity (OR: 4.073, 95% CI: 1.091–15.203). Macula-off retinal detachment (OR: 0.165, 95% CI: 0.033–0.822) and the presence of PVR at the time of presentation (OR: 0.167, 95% CI: 0.042–0.666) were associated with a worse (less than 5/200) postoperative visual acuity. In addition to these variables, the recurring retinal detachments during follow-up were also associated with a worse prognosis (OR: 0.149, 95% CI: 0.037–0.597). These findings are similar to the results of other studies of adult or pediatric retinal detachment [2, 3, 10, 11]. Although these variables are found to be predictors of a worse outcome after retinal detachment surgery, there is no scoring system that incorporates these variables. Furthermore, it was not surprising that patients with endophthalmitis or a suprachoroidal hemorrhage had worse anatomical and functional outcomes in this study. However, the numbers of patients were too limited to draw a statistically significant association for endophthalmitis and suprachoroidal hemorrhage.

The OTS is widely used after open- or closed-globe trauma in adult patients regardless of whether or not the patient presents with retinal detachment. Its prognostic value is well recognized. Some authors recently reported a good predictive value for OTS in pediatric globe injuries [12, 13]. Although RAPD often cannot be evaluated in traumatic children preoperatively, it has been suggested that an adapted OTS that does not consider RAPD has a high predictive value [6]. Initial visual acuity and the presence of RAPD are important factors used to determine the OTS. However, these two measurements are neither easy to obtain nor reliable in children after acute trauma. Thus, Acar et al. [5] suggested that the POTS could serve as an alternative to the OTS. However, when comparing the categorical distribution of final visual acuity in all categories, our results were significantly worse than those suggested by the OTS and POTS. The only study that evaluated the predictive value of the OTS in pediatric patients with retinal detachment was the study of Sarazzin et al. [2], and they reported that the visual acuity in these children was poorer when compared with the expected visual acuity based on the OTS. In agreement with Sarazzin et al. [2], and in contrast to other studies [5–8, 12, 13], we suggest that the OTS and POTS should be used with caution in pediatric patients suffering from retinal detachment.

Because of amblyopia risk, pediatric traumatic retinal detachments are considerably more morbid than adult traumatic retinal detachments. Associated traumatic pathologies, such as corneal lacerations and opacities, crystalline lens pathologies, and irregular astigmatism, also have amblyogenic potential. Accordingly, functional mismatch between anatomical and functional success may (at least partially) result from amblyopia. However, the associated pathologies reported in this study, as well as others like simultaneous traumatic optic neuropathy, may also be important in functional visual loss even in the absence of amblyopia.

In conclusion, this study shows that visual acuity, the presence of macula-off RD, and PVR at the time of presentation and recurrent retinal detachment during follow-up are all important predictors of final visual acuity. Some of the factors associated with a worse outcome are reported in this study and in the literature. However, there is currently no reliable *scoring system* to predict visual acuity in these patients. Although the OTS and POTS take into consideration the presence or absence of a retinal detachment and they are proposed to predict visual acuity after pediatric ocular trauma in patients with or without retinal detachment, they do not have a good predictive value if the patient presents with a retinal detachment.

## Figures and Tables

**Table 1:** Demographics and trauma type.

	Number	Percentage	*p* ^*∗*^
Gender			**<0.001**
Male	47	82.5	
Female	10	17.5	

Age			0.150
0 to <6	13	22.8	
6 to <12	25	43.9	
12 to <18	19	33.3	

Laterality			0.996
Right	29	50.9	
Left	28	49.1	

Trauma type			**0.008**
Open	36	63.2	
Closed	21	36.8	

^*∗*^Chi-square test.

**Table 2:** Mechanism of injury.

	Number	Percent
Knife	12	30
Stone	10	18
Ball	6	11
Projectile (metallic IOFB)	4	7
Falls	3	5
Blunt assault	3	5
Door handle	2	4
Metal wire	2	4
Explosion	2	4
Gun shot	1	2
Thornbush	1	2
Wood	1	2
Plastic bullet	1	2
Glass	1	2
Tweezers	1	2
Corner of a table	1	2
Fork	1	2
Nail	1	2
Pencil	1	2
Broom handle	1	2
Hair clip	1	2
Motor vehicle accident	1	2

**Table 3:** Associated ocular pathologies.

	Number	Percent
Endophthalmitis	2	4
Hyphema	12	21
Iridodialysis/cyclodialysis	2	4
Cataract	22	39
Crystalline lens dislocation	4	7
Intravitreal hemorrhage	11	19
Subretinal hemorrhage	1	2
Suprachoroidal hemorrhage	2	4
Macula-off RD	22	37
PVR	21	37

RD: retinal detachment; PVR: proliferative vitreoretinopathy ≥Grade B.

**Table 4:** Preoperative and postoperative visual acuities.

	Missing	NLP	LP/HM	1/200–19/200	20/200–20/50	≥20/40
	*n* (%)	*n* (%)	*n* (%)	*n* (%)	*n* (%)	*n* (%)
Preoperative	5 (9)	4 (7)	35 (61)	8 (14)	4 (7)	1 (2)
Postoperative	1 (2)	5 (9)	27 (46)	14 (25)	8 (14)	3 (5)

NLP: no light perception; LP: light perception; HM: hand motion; *n*: number.

**Table 5:** Factors associated with anatomical and functional success.

	*n*	Anatomical success	*p*	Functional success	*p*	OR (95% CI)	*p* ^*∗*^
Injury type			0.762		0.572	—	—
Open-globe trauma	36	25/36 (69%)		12/36 (33%)			
Closed-globe trauma	21	16/21 (76%)		9/21 (43%)			

Presenting DCVA			0.472		**0.047**	4.073	**0.031**
>Hand motion	13	11/13 (85%)		8/13 (62%)		(1.091–15.203)	
≤Hand motion	39	27/39 (69%)		11/39 (28%)			

Hyphema			0.287		0.177	—	—
Present	12	7/12 (58%)		2/12 (17%)			
Absent	45	34/45 (76%)		19/45 (42%)			

Crystalline lens dislocation			0.568		1.00	—	—
Present	4	4/4 (%100)		1/4 (25%)			
Absent	53	37/53 (70%)		20/53 (38%)			

Intraocular foreign body			0.660		0.404	—	—
Present	7	6/7 (86%)		4/7 (57%)			
Absent	50	35/50 (70%)		17/50 (34%)			

Intravitreal hemorrhage			1.00		0.511	—	—
Present	11	8/11 (73%)		5/11 (46%)			
Absent	46	33/46 (72%)		16/46 (35%)			

PVR			0.232		**0.010**	0.167	**0.005**
≥Grade B	21	13/21 (62%)		3/21 (14%)		(0.042–0.666)	
<Grade B	36	28/36 (78%)		18/36 (50%)			

Macula-off retinal detachment			0.752		**0.030**	0.165 (0.033–0.822)	**0.012**
Yes	16	11/16 (69%)		2/16 (13%)			
No	41	30/41 (73%)		19/41 (46%)			

Recurrent retinal detachment			0.763		**0.005**	0.149 (0.037–0.597)	**0.003**
Yes	22	15/22 (68%)		3/22 (14%)			
No	35	26/35 (74%)		18/35 (51%)			
Suprachoroidal hemorrhage			0.486		0.526	—	—

Present	2	1/2 (50%)		0/2 (0%)			
Absent	55	40/55 (73%)		21/55 (38%)			

Endophthalmitis			0.486		1.00	—	—
Yes	2	1/2 (50%)		1/2 (50%)			
No	55	40/55 (73%)		20/55 (36%)			

DCVA: distance-corrected visual acuity; PVR: proliferative vitreoretinopathy; OR: odds ratio; CI: confidence interval; chi-square exact test, two-tailed *p* value; ^*∗*^logistic regression *p* value.

**Table 6:** Comparison of final visual acuities and OTS categorical distributions between OTS study and our series.

Sum of raw points	OTS	NLP	LP/HM	1/200–19/200	20/200–20/50	≥20/40	*p*
		*A*/*B*	*A*/*B*	*A*/*B*	*A*/*B*	*A*/*B*	
0–44	1	74/0	15/60	7/20	3/20	1/0	<0.001
45–65	2	27/17	26/60	18/13	15/10	15/0	<0.001
66–80	3	2/0	11/9	15/46	31/27	41/18	<0.001
81–91	4	1/0	2/0	3/0	22/0	73/100	<0.001
92–100	5	0/0	1/0	1/0	57/0	94/0	N/A

OTS: Ocular Trauma Score; NLP: no light perception; LP: light perception; HM: hand motion; *A*: OTS study results (%); *B*: our study results (%); chi-square exact test, two-tailed *p* value.

**Table 7:** Comparison of final visual acuities and POTS categorical distributions between POTS study and our series.

Sum of raw points	POTS	NLP	LP/HM	CF	0.1–0.5	≥0.6	*p*
		*A*/*B*	*A*/*B*	*A*/*B*	*A*/*B*	*A*/*B*	
0–44	1	26/0	11/60	11/20	26/20	26/0	<0.001
45–65	2	0/17	5/60	11/13	26/10	58/0	<0.001
66–80	3	0/0	0/9	0/46	33/27	67/18	<0.001
81–91	4	0/0	0/0	0/0	0/0	100/100	N/A
92–100	5	0/0	0/0	0/0	0/0	100/0	N/A

POTS: Pediatric Ocular Trauma Score; NLP: no light perception; LP: light perception; HM: hand motion; CF: counting fingers; *A*: OTS study results (%); *B*: our study results (%); chi-square exact test, two-tailed *p* value.

## Data Availability

The datasets used and/or analyzed during the current study are available from the corresponding author on reasonable request.
